# Multiple cannulated screw fixation of femoral neck fractures with comminution in young- and middle-aged patients

**DOI:** 10.1186/s13018-022-03157-7

**Published:** 2022-05-18

**Authors:** Zhe Han, Wumti Taxi, Haobo Jia, NengNeng Ji, DongDong Cao, Xiang Sun, Chao Han, Mengqi Xie, Xinlong Ma, Qiang Dong

**Affiliations:** 1 Department of Hip Trauma, Tianjin Hospital, No. 406 Jiefang South Road, Hexi District, Tianjin, 300211 China; 2 Tianjin University of Traditional Chinese Medicine, Tianjin, 301617 China; 3 Institute of Orthopaedics, Tianjin Hospital, Tianjin, 300211 China

**Keywords:** Femoral neck fracture, Comminution, Cannulated screw fixation, Complication

## Abstract

**Objective:**

To investigate the distribution and influence of comminution in femoral neck fracture (FNF) patients after cannulated screw fixation (CSF).

**Methods:**

From January 2019 to June 2020, a total of 473 patients aged 23–65 years with FNF treated by CSF were included in the present study. Based on location of the cortical comminution, FNF patients were assigned to two groups: the comminution group (anterior comminution, posterior comminution, superior comminution, inferior comminution, multiple comminutions) or the without comminution group. The incidence of postoperative complications, quality of life and functional outcomes was recorded at 1-year follow-up.

**Results:**

Comminution was more likely to appear in displaced FNF patients (86.8%) compared with non-displaced FNF patients (8.9%), and the rate of comminution was closely associated with Pauwels classification (3.2% vs 53.5% vs 83.9%, *P* < 0.05). The incidence of osteonecrosis of the femoral head (ONFH, 11.3% vs 2.9%, *P* < 0.05), nonunion (7.5% vs 1.7%, *P* < 0.05), femoral neck shortening (21.6% vs 13.4%, *P* < 0.05) and internal fixation failure (11.8% vs 2.9%, *P* < 0.05) was significantly higher in FNF patients with comminutions, especially with multiple comminutions, than those without. Furthermore, there was a significant difference in the Harris hip score (HHS, 85.6 ± 15.6 vs 91.3 ± 10.8, *P* < 0.05) and EuroQol five dimensions questionnaire (EQ-5D, 0.85 ± 0.17 vs 0.91 ± 0.18, *P* < 0.05) between FNF patients with comminution and those without. There was no significant difference in Visual analogue scale scores (VAS, 1.46 ± 2.49 vs 1.13 ± 1.80, *P* > 0.05) between two groups at 1 year post-surgery.

**Conclusion:**

Comminution is a risk factor for postoperative complications in young- and middle-aged patients with displaced and Pauwels type III FNF who undergo CSF. This can influence the recovery of hip function, thereby impacting quality of life. Further evaluation with a more comprehensive study design, larger sample and long-term follow-up is needed.

## Introduction

Femoral neck fractures (FNFs) are a common injury in both elderly patients as a result of low-energy trauma and younger patients as a result of high-energy trauma. FNFs are considered a serious injury due to the high-risk nature of surgical treatment, particularly in elderly patients with multiple comorbidities who are at higher risk of poor outcomes [1–3]. It has been estimated that the total number of hip fractures will increase to 6.3 million worldwide by 2050, with FNFs accounting for approximately 50% of the total number [4, 5]. The neck of the femur has a complex blood supply and unique biomechanical characteristics, which can result in a series of complications such as nonunion and osteonecrosis of the femoral head (ONFH), a leading cause of pain and dysfunction [6]. These poor outcomes result in high disability rates and health-care resource use, creating a serious socioeconomic burden [5].

Therapeutic strategies for FNFs include either arthroplasty or internal fixation depending on a number of factors, including patient age, fracture pattern and functional requirements. In contrast to the management FNF in elderly patients, multiple cannulated screw fixation (CSF) is a widely accepted approach for the management of FNF in younger patients based on its easy operation, reduced tissue damage and preservation of original joints [7, 8]. The rate of complications including nonunion, ONFH and implant failure of internal fixation following FNFs is 9–30%, resulting in disability and subsequently requiring revision surgery or conversion to arthroplasty procedures at rates of 20–36% [9–11]. Evidence suggests that high failure rate of internal fixation for FNFs is related to inadequate reduction, fracture type, bone quality and loosening of fixation [10–12].

An increasing number of studies indicate that disruption to the cortex of the femoral neck is common in patients with FNFs. For example, Collinge et al. found that 96% of patients with vertical shear FNF had comminutions in the inferior (94%) and posterior (82%) femoral neck [13]; Huang et al. found that 36.5% of displaced FNF patients had a disrupted posterior cortex, acting as an independent risk factor for postoperative ONFH, union and shortening [6]. Several investigations also show that comminution of the femoral neck is a serious risk factor for loss of cortical support of cannulated screws, resulting in internal fixation failure even when adequate reduction is achieved [7, 12]. Therefore, the integrity of the cortex of femoral neck is believed to be an important factor for successful surgical management of patients of all ages with FNF.

However, the distribution and influence of comminutions in the femoral neck in FNF patients with CSF remains largely unknown. The goals of present study are to discuss the characteristics of cortical defect in the femoral neck of FNF patients and determine whether there is a correlation between comminution distribution and prognosis. A more robust understanding of these findings will improve understanding of the FNF injury mechanism of FNF and may improve treatment strategies and reduce postoperative complications.

## Materials and methods

### Participants

**Fig. 1:**
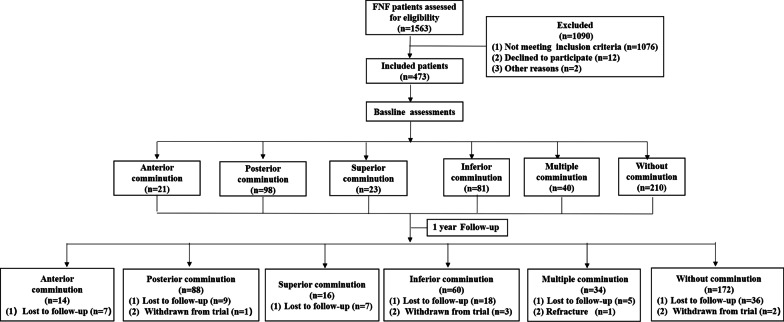
Flowchart of patients in 6 groups. FNF: femoral neck fracture

This retrospective cohort study included patients with FNFs in Tianjin Hospital from January 2019 to June 2020. All methods were conducted in accordance with the Helsinki Declaration and approved by the Ethics Committee of Tianjin Hospital. All participants were fully aware of the nature, purpose, procedures and risks of the study and provided written informed consent. The flowchart of participants is shown in Fig. 1.

### The inclusion and exclusion criteria

Inclusion criteria include: (1) age ranging from 18 to 65 years old; (2) with a unilateral FNF; (3) underwent multiple CSF and achieved acceptable reduction quality; (4) completed 1-year follow-up; and (5) consent to be included in the study.

Exclusion criteria include: (1) pathological fracture or old fracture (more than 14 days); (2) previous ipsilateral hip diseases; (3) bilateral FNF or combined with femoral shaft fracture; (4) a bone metabolism disorder; (5) the occurrence of diseases affecting the function of lower limbs, death or no follow-up; (6) serious nervous system or cognitive impairment such as dementia or Parkinson disease; (7) pregnant or lactating women; and (8) incomplete clinical data.

### Radiographic assessment and grouping

**Fig. 2:**
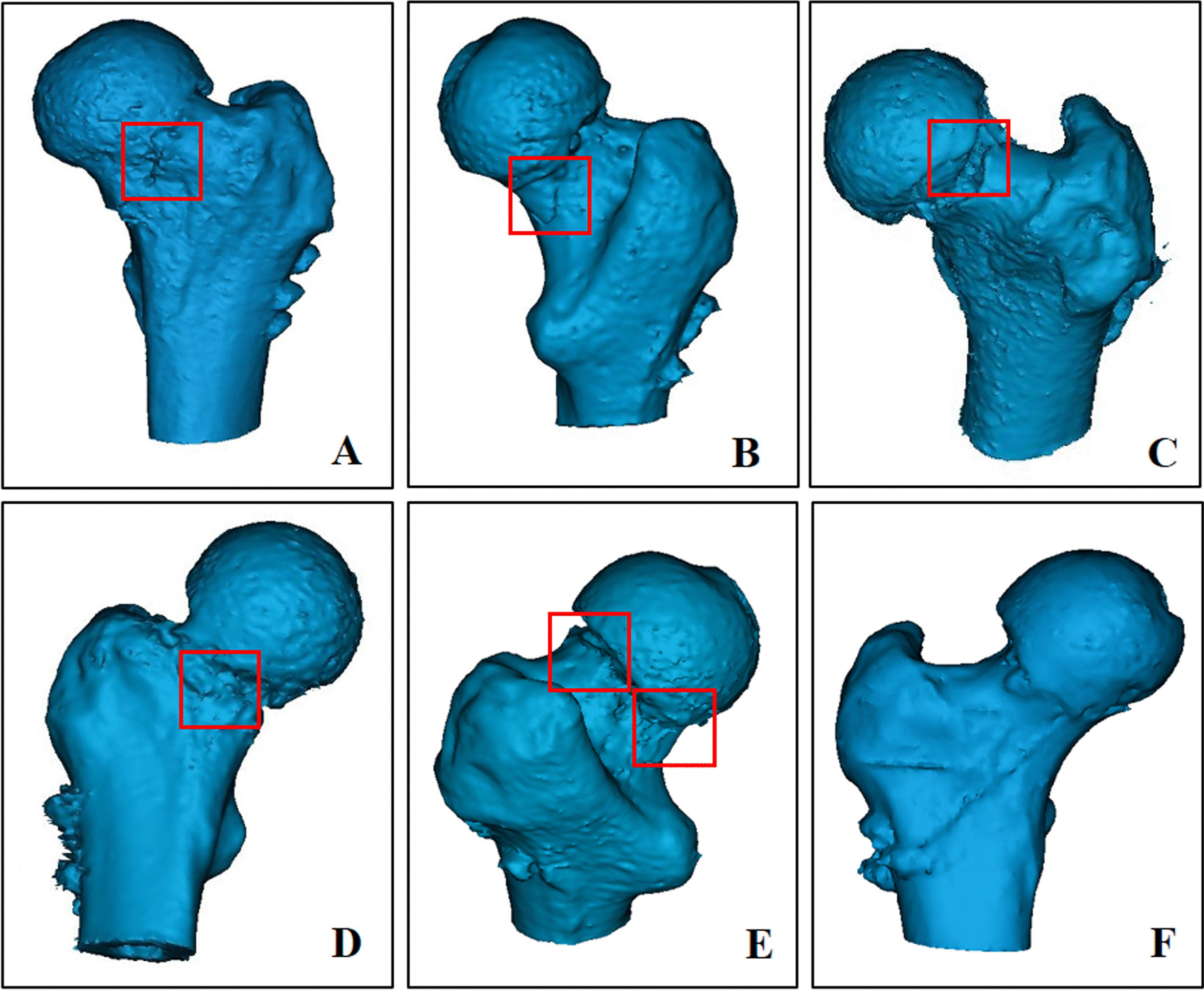
The 3D-CT showing that different distribution of comminutions in femoral neck on FNF patients. **A** anterior comminution; **B** posterior comminution; **C** superior comminution; **D** inferior comminution; **E** multiple comminutions; and **F** without comminution

Anteroposterior (AP) radiographs were employed to estimate fractures based on the Garden classification [14] and Pauwels classification [15]. Postoperative three-dimensional computed tomography (3D-CT) reconstruction was performed to evaluate the existence and location of comminution in the femoral neck (Fig. 2). All imaging was separately reviewed, and morphology was assessed by two radiologists and two orthopedic trauma surgeons. All disagreements were resolved by discussion. Patients were divided into 6 groups according to the location of comminution in the femoral neck: (1) anterior comminution; (2) posterior comminution; (3) superior comminution; (4) inferior comminution; (5) multiple comminutions; and (6) without comminution.

### Operative and postoperative procedures

Closed reduction or open reduction with three standard CSFs of FNFs was carried out in all patients under either general or epidural anesthesia. The reduction quality (excellent, good and fair) of the FNFs was considered to be acceptable according to that described by Haidukewych et al. [16]. Patients were encouraged to rehabilitate in bed immediately after surgery with non-weight-bearing mobilization over 8 weeks, weight bearing was allowed with walker/crutches once callus formation was verified using radiography, and full weight bearing was allowed depending on fracture healing at monthly postoperative re-examination.

### Follow-up and outcome measurement

After discharge, patients were required to go to the special follow-up clinic once a month until the fracture was healed. After the fracture healed, patients were rechecked at 1 year post-op, which included radiographic and clinical examinations and surveyed for information on complications, quality of life, pain and hip function. If the participant could not attend the clinic for re-examination, they were asked to undergo X-ray imaging at local hospital and e-mail images to the surgeons. Finally, primary and secondary outcomes were measured at 1 year post-op.

Primary outcomes were postoperative complications including nonunion, ONFH, shortening and fixation failure. Fracture nonunion was defined as a clear, visible fracture line or the absence of bridging cortical bone at 6 months postoperative, which was evaluated by imaging examination [17, 18]. ONFH was identified using magnetic resonance imaging (MRI) or radiography, following the radiological criteria by Arlet et al. [19]. According to the description by Zlowodzki et al. [20], the length of femoral neck was measured and femoral neck shortening was defined as a shortening length exceeding 5 mm in immediate postoperative film compared with last follow-up [20]. Internal fixation failure was evaluated by radiography, including screw withdraw, implant cut out, screw loose, varus deformity > 10° and re-displacement > 5 mm.

Secondary outcomes were the Harris hip score (HHS), EQ-5D (EuroQol five dimensions questionnaire) score and visual analogue scale score (VAS) at 1 year post-op. HHS was widely used to evaluate hip function, including four aspects: pain (1item, 0–44 points), function (7 items, 0–47 points), deformity (1 item, 0–4 points) and range of activity (2 items, 0–5 points) [21]. EQ-5D, developed by the EuroQol team, is a patient reporting tool to measure quality of life [22]. The questionnaire contains five questions covering five different aspects including mobility, self-care, daily activities, pain/discomfort and anxiety/depression, and a specific value set for Chinese patients obtained by Luo et al. [23]. VAS (score from 0 to 10) evaluates pain intensity on a scale where 0 is painless; less than 3 is mild pain that the patient can endure; 4–6 is pain that the patient can bear and can sleep; and 7–10 is severe pain that the patient cannot bear [24].

### Statistical analysis

All statistical analyses were performed by Statistical Package for Social Sciences (SPSS) version 25.0 (SPSS Inc., Chicago, IL, USA). The categorical variables of clinical data and outcomes were assessed using Pearson’s Chi-squared test or Fisher’s exact test. The continuous variable data were assessed for a fit to a normal distribution and for homogeneity of variance using the Shapiro–Wilk test and Bartlett test, which was represented as mean ± SD. Student’s *t* test or the analysis of variance (ANOVA) was used for inter-group comparisons. *P* value less than 0.05 was considered statistically significant.

## Results

A total of 473 patients with FNFs were identified from January 2019 to June 2020, 82 patients were lost to follow-up, 6 patients declined participation, and 1 patient dropped out due to re-fracture. A total of 384 patients completed the 1-year follow-up and were included in this study. Of those patients, 14 had anterior comminution, 88 had posterior comminution, 16 had superior comminution, 60 had inferior comminution, 34 had multiple comminutions, and 172 patients did not have comminution (Fig. 1). There was no significant difference in regard to age, sex, body mass index (BMI), injury mechanism, American Society of Anesthesiologists (ASA) score, from initial injury to operation, operative time, duration of hospitalization and follow-up time among the groups (*P* > 0.05, Table 1). However, intraoperative blood loss and open reduction rate were increased in the multiple comminution group compared with other groups (*P* < 0.05, Table 1).

**Table 1:** Characteristics of participants

Variables	Anterior comminution	Posterior comminution	Superior comminution	Inferior comminution	Multiple comminution	Without comminution	*P* value
	(*n* = 14)	(*n* = 88)	(*n* = 16)	(*n* = 60)	(*n* = 34)	(*n* = 172)	
Age (years)	51.7 ± 9.6	52.1 ± 7.3	51.5 ± 7.3	49.6 ± 10.3	49.9 ± 9.6	49.3 ± 13.7	0.515
Female (%)	6 (42.9)	44 (50.0)	7 (44.8)	22 (36.7)	12 (35.3)	93 (54.1)	0.144
BMI (Kg/m^2^)	23.1 ± 3.8	23.5 ± 4.3	22.8 ± 4.1	23.9 ± 4.7	23.2 ± 4.6	22.9 ± 3.7	0.659
Tobacco use (%)	3 (21.4)	18 (20.4)	4 (25.0)	10 (16.6)	8 (23.5)	32 (18.6)	0.951
Alcohol use (%)	2 (14.3)	12 (13.6)	1 (6.0)	7 (11.7)	6 (17.6)	25 (14.5)	0.913
Mechanism of injury (%)							0.800
Traffic accident	5 (35.7)	32 (36.4)	7 (43.8)	23(38.3)	15(44.1)	45(26.2)	0.505
Fall	9 (56.3)	54 (61.4)	9 (56.3)	35 (58.4)	18 (52.9)	124 (72.1)	
Others	0 (0.0)	2 (2.2)	0 (0.0)	2 (3.3)	1 (3.0)	3 (1.7)	
ASA class (%)							0.511
ASA I/II	12 (85.7)	82 (93.2)	14 (87.5)	53 (88.3)	30 (88.2)	160 (93.0)	
ASA III/IV	2 (14.3)	6 (6.8)	2 (12.5)	7 (11.6)	4 (11.8)	12 (7.0)	
Reduction methods (%)							
CRIF	12 (85.7)	63 (71.6)	14 (87.5)	53 (88.3)	22 (64.7)*	159 (92.4)	< 0.001
ORIF	2 (14.3)	15(28.4)	2 (12.5)	7 (11.7)	12 (35.3)*	13 (7.6)	
Time to surgery (day)	2.6 ± 1.0	2.5 ± 0.9	2.4 ± 1.1	2.6 ± 0.9	2.4 ± 0.8	2.7 ± 0.9	0.347
Operation time(min)	48.8 ± 15.8	49.2 ± 18.4	47.5 ± 14.1	50.2 ± 20.0	55.1 ± 19.2	45.8 ± 16.7	0.102
Intraoperative							
blood loss (ml)	85.4 ± 55.1	83.7 ± 53.9	79.2 ± 47.5	80.6 ± 53.3	123.5 ± 82.4*	88.6 ± 51.2	< 0.001
Length of hospital stay (day)	5.6 ± 2.3	5.8 ± 1.9	6.0 ± 1.9	5.7 ± 2.2	5.5 ± 2.4	5.6 ± 2.0	0.942
Follow-up time (years)	1.6 ± 0.4	1.5 ± 0.3	1.6 ± 0.2	1.6 ± 0.3	1.5 ± 0.3	1.6 ± 0.4	0.237

CRIF, Closed reduction and internal fixation; ORIF, open reduction and internal fixation

*Difference versus other groups (*P* < 0.05)

To analyze the morphological characteristics of the cortical defect in the femoral neck, we surveyed the relationship between fracture type and comminution distribution. As shown in Table 2, for Garden classification, the proportion of comminution in displaced FNFs was significantly higher than that in non-displaced FNFs (*P* < 0.05). The comminution was concentrated on the posterior and inferior of the femoral neck in both non-displaced and displaced FNFs (*P* < 0.05), while there was no difference in comminution distribution between the two types of fractures (*P* > 0.05). The amount of comminution increased significantly with an increase in each Pauwels classification type (*P* < 0.05). The incidence of posterior and inferior comminution was higher than the other groups, and there was no difference in comminution distribution in different fracture patterns (*P* > 0.05).

**Table 2:** The relationship between fracture types and location of comminution

Variables	With comminution (*n* = 212)					Without comminution (n = 172)	*P* value
	Anterior comminution (*n* = 14)	Posterior comminution (*n* = 88)	Superior comminution (*n* = 16)	Inferior comminution (*n* = 60)	Multiple comminution (*n* = 34)		
*Garden classification*							< 0.001
No-displacement fracture (Garden I/II)	14 (8.9)					142 (91.1)	0.063
	0 (0.0)	6 (3.8)	0 (0.0)	8 (5.1)	0 (0.0)		
Displacement fractures (Garden III/IV)	198 (86.8)					30 (13.2)	
	14 (6.1)	82 (36.0)	16 (7.0)	52 (22.8)	34 (14.9)		
*Pauwels classification*							< 0.001
Type I	2 (3.2)					60 (96.8)	0.330
	0 (0.0)	0 (0.0)	0 (0.0)	2 (3.2)	0 (0.0)		
Type II	106 (53.5)					92 (46.5)	
	8 (4.0)	44 (22.2)	6 (3.0)	34 (17.2)	14 (7.1)		
Type III	104 (83.9					20 (16.1)	
	6 (4.8)	44 (35.5)	10 (8.1)	24 (19.4)	20 (16.1)		

As illustrated in Table 3, the occurrence of postoperative complications, including nonunion, ONFH, shortening and fixation failure, was significantly higher in the comminution group than those without comminution (*P* < 0.05). As demonstrated by further inter-group analysis, the incidence of postoperative complications in FNF with multiple comminutions was significantly higher than those in the other four groups (*P* < 0.05). There was no clear correlation between a single comminution in the femoral neck and postoperative complications (*P* > 0.05).

**Table 3:** Comparison of primary outcomes in different groups

Variables	With comminution (*n* = 212)					Without comminution (*n* = 172)	*P* value
	Anterior comminution (*n* = 14)	Posterior comminution (*n* = 88)	Superior comminution (*n* = 16)	Inferior comminution (*n* = 60)	Multiple comminution (*n* = 34)		
Nonunion (%)	16 (7.5)					3 (1.7)	0.009
	1 (7.1)	6 (6.8)	0 (0.0)	4 (6.7)	5 (14.7)*		
ONFH (%)	24 (11.3)					5 (2.9)	0.002
	0 (0.0)	10 (11.3)	0 (0.0)	5 (8.3)	9 (26.4)*		
Shortening (%)	46 (21.6)					23 (13.4)	0.044
	2 (14.2)	18 (20.4)	2 (12.5)	10 (16.6)	14 (41.2)*		
IF failure (%)	19 (11.8)					5 (2.9)	0.020
	0 (0.0)	6 (6.8)	1 (6.2)	4 (6.6)	8 (23.5)*		

ONFH, Osteonecrosis of the femoral head; IF, internal fixation

^*^Difference versus other groups with comminution (*P* < 0.05)

The HHS, EQ-5D index and VAS score were measured in patients at 1 year postoperative. As displayed in Table 4, there was a significant difference in the HHS and EQ-5D index between the FNF with comminution group and the FNF without comminution group (*P* < 0.05). However, there was no difference in VAS scores between the FNF with comminution group and FNF without comminution group (*P* > 0.05). There was no close relationship between secondary outcomes and FNF patients with different comminution locations 1 year after surgery (*P* > 0.05).

**Table 4:** Comparison of secondary outcomes in different groups

Variables	With comminution (n = 212)					Without comminution (*n* = 172)	*P* value
	Anterior comminution (*n* = 14)	Posterior comminution (*n* = 88)	Superior comminution (*n* = 16)	Inferior comminution (*n* = 60)	Multiple comminution (*n* = 34)		
HHS	85.6 ± 15.6					91.3 ± 10.8	< 0.001
	89.3 ± 6.5	84.5 ± 15.3	87.2 ± 13.9	88.1 ± 16.1	81.7 ± 18.0		
EQ-5D	0.85 ± 0.17					0.91 ± 0.18	< 0.001
	0.89 ± 0.10	0.84 ± 0.19	0.85 ± 0.13	0.87 ± 0.18	0.82 ± 0.16		
VAS score	1.46 ± 2.49					1.13 ± 1.80	0.146
	1.15 ± 1.23	1.51 ± 2.89	1.37 ± 1.86	1.41 ± 2.25	1.59 ± 2.46		

Results are expressed as mean ± SD; *HSS* Harris hip score, *VAS* visual analogue scale

## Discussion

The incidence of FNF in young- and middle-aged people is growing as a result of high-energy traumas, resulting in impacts to quality of life, hip function and labor capacity, thereby creating an economic burden to the global society [25, 26]. Despite advances in head-preserving surgical techniques and implants for FNF, the high rates of postoperative osteonecrosis, nonunion and femur shortening are problematic and challenging for young and active adults [27, 28]. It is commonly believed that satisfactory reduction, accurate positioning and placement of the implant and appropriate rehabilitation are the critical to achieve fracture healing and recovery of function, in addition to preventing varus collapse and ONFH [10–12]. Cortical bone deficiency in the femoral neck is considered an important factor for biological healing and biomechanical stability for FNF with internal fixation [7, 29]. Evidence has shown that 50–96% of FNF patients have femoral neck comminution, most of which is posterior comminution with the incidence between 50 and 82% [6, 13, 30–32]. This may be explained because young- and middle-aged people with FNF are usually subjected to high-energy trauma, which exceeds the stress that the femoral neck can bear, resulting in the comminuting of the bone. In the elderly population, the cortex of femoral neck is thicker and often more frail, resulting in easier comminution in the femoral neck as a result of low-energy trauma. FNFs with comminution are more unstable [36]; therefore, we aim to explore the relationship between morphological characteristics of femoral neck deficiencies and prognosis to improve the therapeutic strategies for future patients with similar injuries.

To our knowledge, this is the first systematic study to describe the distribution of cortical comminution in patients with FNF. Similar to previous studies, our results showed that cortical comminution existed in displaced FNFs compared with non-displaced FNFs, while posterior comminution was the most common type of cortical deficiency in the femoral neck. The comminution mechanism of the posterior cortex in FNFs had been described in different ways. Garden et al. [33] believed that extreme external rotation of the distal fragment would impact the fragile posterior cortical shell near the junction with the head fragment, leading to posterior comminution and fracture, resulting in an intracapsular fracture. Kocher et al. [34] indicated that in the course of the injury, at the moment of vertical trauma in the femoral neck simultaneous with external rotation of the limb, the femoral head is restricted by the iliofemoral ligament and capsule and impinged by acetabulum’s posterior ring, leading to posterior cortical comminution. Additionally, several studies suggest regional cortical comminution is related to the characteristics of cortical bone structure in the femoral neck. For example, because the cortex of femoral neck is thinner from the anterior inferior to the posterior, the greatest cortical damage would be in the posterior side when the femoral neck is subjected to trauma and impact [35, 36]. Unexpectedly, our results found that inferior comminution was the second most common in FNF patients, which has not been reported before. We suggest the following injury mechanism of FNF with inferior comminution: When the proximal femur suffers both vertical traumas with varus, stress concentration would be in the inferior femoral neck, subsequently causing cortical comminution; the inferior cortex in the femoral neck is thinner where the cortical damage occurs in this weak area when the femoral neck subjected to trauma and impact.

Furthermore, our study explored the relationship between Pauwels classification type and distribution of cortical comminutions in FNF patients. Surprisingly, the incidence rate of cortical comminution increased positively with fracture types. Therefore, our findings suggest that in vertical FNFs, not only higher-degree lesions but also cortical deficiency worsens instability, progressive fracture displacement and risk of varus collapse, resulting in serious postoperative complications and fixation failure.

ONFH is a common and serious complication after internal fixation of FNFs, especially in young patients [37]. Incidence in this population is as high as 8.9% [38]. A great deal of evidence indicates that the occurrence of ONFH is the result of interruption of the femoral head blood supply due to traumatic vascular injury or insufficient blood supply caused by the increase of intracapsular pressure after fracture. Another potential cause of ONFH is stress on the femoral head caused by instability of the normal structure of the proximal femur, resulting excessive physiological load and mechanical destruction of trabeculae bone, leading to collapse [39–41]. Increasing evidence shows that that cortical comminution is a factor in ONFH in FNF patients after surgery. For example, Scheck et al. found posterior comminution increased the risk of postoperative ONFH in FNF patients [29]; Huang et al. found that ONFH after internal fixation in FNF patients with posterior cortical comminution was 34.2%, which was much higher than that in patients without comminution (4.8%) [6]. Similar to previous studies, our findings show that the incidence of ONFH in FNF patients with comminution was significantly higher than those without comminution. In particular, FNF patients with multiple comminutions had a much higher risk of ONFH compared to those with a single comminution. There was no significant correlation between the location of the comminution and rate of ONFH. There is a paucity of research investigating the influence of cortical deficiency on FNFs after internal fixation. We propose the following mechanism caused by cortical comminution in the femoral neck: The destruction of the cortex in the femoral neck in FNF patients, particularly the posterior cortex, can severely disrupt the blood supply to the femoral head, regardless of whether the reduction was restored or not, leading to ONFH. Previous reports have found that initial displacement and posterior comminution are closely associated with femoral neck blood vessel damage; at the same time, increased fracture lines and comminutions can lead to devastating damage in the lateral epiphyseal artery and posterior retinacular vessels, a risk factor in femoral head collapse [42, 43]; and some studies have found that cortical defects are a factor in mechanical instability after internal fixation of FNFs [44], which can impact the distribution of surface stress in the femoral head, particularly in weight-bearing areas, contributing to trabecular microfracture and collapse. Additionally, in clinical practice, it is difficult to reset FNF with comminutions by close reduction, and therefore, open reduction is regarded as necessary to achieve a quality or anatomic reduction [45]. However, anterior and anterolateral approaches during open reduction can lead to lateral femoral circumflex artery injury, retinacular vessels traversing disruption and the periosteum and capsular reflections [46], which can increase the risk of ONFH. Blood vessel damage, instability and increased open reduction rate in FNF patient with multiple comminutions were the main reasons for postoperative ONFH.

The prognosis of femoral neck fractures can be affected by several serious conditions, one of the most common being nonunion [47]. The rate nonunion is between 10 and 30%, a complication which can result in functional disability and require revision surgery [27, 38, 48]. An increasing number of studies have identified that cortical deficiency is a critical factor in nonunion in FNFs. For example, Huang et al. found that posterior comminution in FNFs reduced axial load resistance, resulting in early fracture re-displacement and nonunion [6]; Rawall et al. highlighted that young patients with significant posterior comminution were found to have higher nonunion rates [49]. Similarly, our results show that nonunion rates in FNF patients with comminution were significantly higher than that of FNF patients without comminution (7.5% vs 1.7%), while nonunion rate of FNF patients with multiple comminutions (14.7%) was significantly higher in comparison with FNF patients with a single comminution. Posteromedial comminution of the femoral neck is considered an important factor in fracture stability [29]. According to biomechanical findings, the decline in contact stability between internal fixation and the cortex can happen in FNFs with comminution, leading to rotation of the fragments and initial displacement, resulting in nonunion and osteosynthetic failure [50, 51]. Our results found that the number of internal fixation failures in FNF patients with comminution was more than that in FNF without comminution. Similarly, FNF patients with multiple comminutions had the highest incidence of internal fixation failure (23.5%). Previous studies [13, 50, 52] investigating whether or not anatomical reduction was achieved show that comminutions can create difficulty reconstituting a bony buttress and potentially return to the position of injury, causing internal fixation failure. Additionally, the comminutions can produce a mechanical environment where implants can lose fixed-angle properties, resulting in the fracture site bearing more axial and torsion load creating an ineffective buttress against varus re-displacement.

Increasingly strong evidence suggests that femoral neck shortening occurs as much as 32–65.7% in FNF patients after internal fixation, contributing to gait disorder, hip dysfunction and decline in quality of life [53, 54]. Huang et al. found that the incidence of femoral neck shortening in FNF patients with comminution was significantly higher than that in patients without comminution [6]. In accordance with conclusion of Zlowodzki et al.[20], our study found that the incidence of femoral neck shortening was 18.0% in all cases, and the incidence neck shortening in FNF patients with a disrupted cortex was much higher (21.6%) than that in FNF patients without a disrupted cortex (13.4%). We suggest that cortical deficiency in the femoral neck can impact stability in fracture sites after reduction, causing fragments to slide along the implant allowing impact at the fracture site, particularly when patients experience axial loading during early weight bearing.

The functional outcomes and health-related quality of life indicators in FNF patients with comminution have not been well studied. In our study, we found that HSS and EQ-5D index were significantly worse in patients with comminutions than those without comminution, whereas there were no differences in VAS scores between the two groups. FNF patients with multiple comminutions had worse HSS, EQ-5D index and VAS scores in comparison with those with a single comminution. In our view, higher incidence of ONFH, nonunion and femoral neck shortening in FNF patients with multiple comminutions can have a significant influence on operative recovery of hip function, leading to poor function scores.

Limitations of this study include its retrospective design, short follow-up duration, monocentric analysis with a local population and small sample size. A relatively high proportion of patients did not complete the follow-up (89 out of 473 patients, 18.8%), with the main reasons being change of contact information, incomplete imaging and refusal to participate, which may have influenced our data collection. Finally, although we authenticated fracture morphology using 3D-CT scan, there was no clear definition or description for characteristics of cortical comminution in the femoral neck, resulting in inaccurate grouping of fractures and potential for bias. Larger, multicenter and longer duration randomized studies may help to address these limitations.

## Conclusion

In present study, we found that comminution in the femoral neck commonly occurred in displaced FNFs compared to non-displaced FNFs, with posterior comminution and inferior comminution the most common patterns. As hypothesized, comminution in the femoral neck, especially multiple comminutions, was a risk factor for postoperative ONFH, nonunion, femoral neck shortening and internal fixation failure in young- and middle-aged patients, consequently influencing recovery of hip function and quality of life. We hope that these findings highlight the importance of appropriate FNF management in order to avoid unnecessarily postoperative complications and promote functional recovery in patients.

## Data Availability

Not applicable.

## Abbreviations

FNF1: Femoral neck fracture
CSF: Patients after cannulated screw fixation
HHS: Harris hip score
EQ-5D: EuroQol five dimensions questionnaire
VAS: Visual analogue scale
ONFH: Osteonecrosis of femoral head
AP: Anteroposterior
3D-CT: Three-dimensional computed tomography
MRI: Magnetic resonance imaging;
SPSS: Statistical package for social sciences
ANOVA: Analysis of variance
BMI: Body mass index
ASA: American society of anesthesiologists
